# Sex Alters Hypoxic Responses in *Caenorhabditis elegans*

**DOI:** 10.17912/micropub.biology.002042

**Published:** 2026-03-13

**Authors:** Andrew P. Bischer, Katherine E. Neyland, Nada Ahmed Selim, Andrew P. Wojtovich

**Affiliations:** 1 Department of Anesthesiology and Perioperative Medicine, University of Rochester Medical Center, Rochester, NY, US; 2 Department of Pharmacology and Physiology, University of Rochester Medical Center, Rochester, NY, US

## Abstract

The expression of shared behaviors can exhibit sexual dimorphism, which is often mediated by neuronal sex. However, the role of behavioral entrainment by environmental conditions as a function of underlying sex has been less studied. Here, we demonstrate that *Caenorhabditis elegans* males are more resistant to long-term hypoxic injury than hermaphrodites and that their locomotory speed exhibits reduced sensitivity to acute changes in oxygen. Using cell-specific sex reversal, we investigated whether neuronal biological sex influences oxygen-dependent locomotory behavior. These data suggest that the overexpression of sex determination pathway components can modify hypoxic behavioral responses independently of underlying biological sex.

**Figure 1. Sex influences responses to hypoxia.**
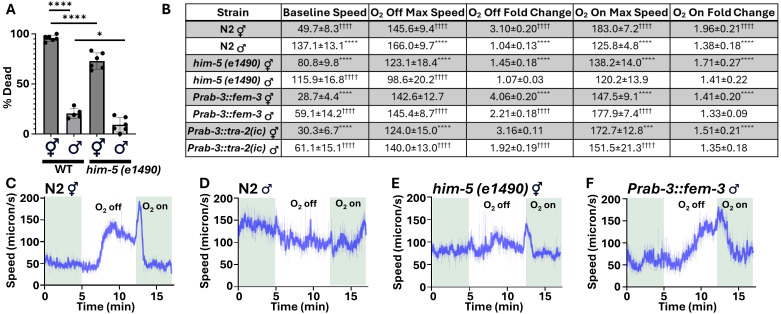
(A) Male (♂) *C. elegans* exhibit greater resistance to hypoxic stress compared to hermaphrodites (⚥). Worms were exposed to hypoxia for 18.5 hours, followed by 24 hours of reoxygenation and recovery. Each data point represents 50-60 worms. * *p* < 0.0332; **** *p* < 0.0001, calculated using one-way ANOVA with Tukey's multiple comparisons test. (B) The table displaying peak speeds during hypoxia (O _2_ off) and reoxygenation (O _2_ on) in O _2_ off/on experiments.&nbsp; Values represent averages from 4 replicates, each containing 50-75 worms. Baseline speed refers to the average speed observed during the 5-minute baseline, measured in microns/second. Maximum speed refers to the peak speed observed, measured in microns/second. Fold change indicates the difference between the base of the peak speed and the maximum speed. Data includes standard deviation. *** *p* < 0.001 compared to N2 ⚥, **** *p* < 0.0001 compared to N2 ⚥; †††† *p* < 0.0001 compared to N2 ♂, calculated using two-way ANOVA with Tukey's multiple comparisons test. (C) O _2_ off/on response of N2 hermaphrodites. The graph represents data from 4 replicates, with 50-75 worms per replicate. (D) O _2_ off/on response of N2 males. Graph represents data from 4 replicates, with 50-75 worms per replicate. (E-F) Representative graphs of disrupted O _2_ off/on for hermaphrodites (E, *him-5* ) and males (F, *Prab-3:: fem-3* ).

## Description

*Caenorhabditis elegans* exhibits sex-specific responses to a variety of external stimuli, including food and pheromones (Lawson et al., 2020). Many of these processes are coordinated by neurons and result in distinct behavioral outcomes (Aprison & Ruvinsky, 2019; Whittaker & Sternberg, 2009). Oxygen levels in soil can vary, and *C. elegans* are attracted to oxygen concentrations between 5-12%, actively avoiding levels above and below the preferred range (Gray et al., 2004). Both hypoxia and hyperoxia are detected neuronally, with distinct signaling cascades mediating the response to each condition (Chang & Bargmann, 2008). Males and hermaphrodites of *C. elegans* exhibit differences in their responses to external cues, such as food (Wexler et al., 2020). To investigate whether genetic sex, XX for hermaphrodites and XO for males, influences outcomes following long-term hypoxic exposure, we conducted experiments using day 1 adult worms. Worms were placed in a hypoxic chamber for 18.5 hours, followed by a 24-hour recovery period under normoxic conditions. Prior research indicated that males are more resistant to long-term oxygen deprivation and recovery compared to hermaphrodites (Mendenhall et al., 2009; Padilla et al., 2002). Our results corroborate these findings, showing that hermaphrodites are more susceptible to hypoxic injury than males ( Fig. 1A ). Under these conditions, approximately 80% of male worms survived, compared to under 10% of hermaphrodites. Males naturally occur at low frequencies in *C. elegans* populations. To increase the number of males, we utilized a mutation in *him-5* , which increases nondisjunction of the X chromosome, thereby raising the frequency of males (Hodgkin et al., 1979). Interestingly, we found that the *him-5* mutation alone conferred protection against long-term hypoxic insults in both males and hermaphrodites ( Fig. 1A ). This suggests that *him-5* may influence mechanisms beyond chromosomal disjunction. In summary, our findings demonstrate that males are more resilient than hermaphrodites in response to long-term hypoxia, and the *him-5* mutation provides additional protection against long-term hypoxic exposure.

&nbsp;

We next examined the effect of genetic sex on behavioral changes in response to acute oxygen changes. Previous studies have shown that hermaphrodites respond to hypoxia with a rapid increase in speed, which gradually decreases over time. Upon reoxygenation, hermaphrodites exhibit another increase in speed (Ma et al., 2013; Ma et al., 2012). This behavior is referred to as the O _2_ off and O _2_ on response. To investigate this phenomenon, we measured worms' baseline speed under normoxic conditions for 5 minutes, followed by exposure to nitrogen gas for 7 minutes, and then reoxygenation with air for 5 minutes. Worm speed was tracked using Wormtracker software (Ramot et al., 2008), which allowed for the calculation of individual worm's speed. To quantify the O _2_ off and O _2_ on responses, we calculated the fold change in speed from the maximal to the minimum (base of peak). Our results confirm previous findings: hermaphrodites exhibited a greater than 3-fold increase in speed during the O _2_ off response and nearly a 2-fold increase in speed during the O _2_ on response ( Fig. 1B, C). In contrast, males did not exhibit any significant response to changes in oxygen levels and showed no O _2_ off or O _2_ on response ( Fig. 1B, D). It remains unclear whether the lack of a response in males is due to an inability to sense and react to oxygen fluctuations or if their baseline speed represents their maximal speed. Notably, males have been reported to achieve maximal speeds of 200 microns/second (Suo et al., 2019).

&nbsp;

Next, we tested if *him-5* , a genetic mutation commonly used to increase male populations, affected the ability of hermaphrodites and males to sense changes in oxygen. We found that *him-5* increased the baseline speed and reduced the O _2_ off response in hermaphrodites ( Fig. 1B, E). However, *him-5* did not appear to affect males, as *their* O _2_ off and O _2_ on responses remained similar to those of wild-type males ( Fig. 1B ). These results suggest that the *him-5* mutation may have off-target effects, particularly in hermaphrodites, and highlight the importance of comparing experimental results to a wildtype strain.

&nbsp;

*C. elegans* sense oxygen through neuron-mediated processes, with neurons coordinating behavioral changes (Chang & Bargmann, 2008; Zimmer et al., 2009). To investigate whether the biological sex of the neurons could replicate behavior determined by the genetic sex of the organism, we tested strains that overexpressed the transcription factors *fem-3* and *tra-2* (Fagan et al., 2018; Luo et al., 2024) in neurons. Biological sex can be altered in select tissues by expressing transcription factors (such as *fem-3* and *tra-2* ) that are involved in sex determination without changes in genetic sex. Neuronal masculinization ( *Prab-3:: fem-3* ) in hermaphrodites increased the O _2_ off response but decreased the O _2_ on response compared to N2 hermaphrodites. However, both responses were higher than those observed in N2 males ( Fig. 1B ). Males overexpressing *fem-3* in neurons also exhibited altered behavior, showing increased speed in response to hypoxia (O _2_ off) and subsequent reoxygenation (O _2_ on) ( Fig. 1F ). Similarly, feminization of neurons ( *Prab-3:: tra-2 (ic)* ; constitutively active, intracellular domain) produced comparable results, with overexpression of *tra-2 (ic)* altering behavior in both hermaphrodites and males. Hermaphrodites with feminized neurons displayed a diminished O _2_ on response compared to N2 hermaphrodites ( Fig. 1B ). In males with feminized neurons, the O _2_ off and O _2_ on responses were present but reduced compared to N2 hermaphrodites ( Fig. 1B ). These finding suggest that altering the biological sex of neurons impacts oxygen-sensing behavior, but the responses differ depending on the genetic sex of the organism.

&nbsp;

Our findings underscore the complexity of oxygen sensing and its relationship with neuronal biological sex. &nbsp;Altering the biological sex of neurons can lead to unintended consequences, complicating interpretation of results. Hermaphrodites and males exhibit distinct responses to acute and long-term changes in oxygen concentration. For instance, males demonstrate resistance to long-term hypoxia, suggesting an ability to sense oxygen changes and signal protection mechanisms. However, they lack an O _2_ off/on response, which may point to an intrinsic resistance to hypoxia, potentially linked to lower metabolic demands compared to hermaphrodites. Interestingly, altering neuronal biological sex alone was not sufficient to replicate the behaviors observed in either males or hermaphrodites, indicating that the response to oxygen likely involved input from multiple tissues working in concert. Overexpression of the transcription factor *fem-3* and *tra-2 (ic)* affected worm behavior regardless of sex, suggesting that these transcription factors alone can influence behavior. Furthermore, the biological sex of neurons has been shown to be dynamic under certain conditions, such as nutrient stress. Overexpression of transcription factors like *tra-2 (ic)* and *fem-3* may disrupt the dynamic regulation required for appropriate behavioral responses (Lawson et al., 2020). Timing and expression levels of these transcription factors also appear to play a critical role in sex-based behavioral differences. Overall, our results highlight that biological sex significantly influences behavior in response to oxygen. However, altering neuronal biological sex alone is insufficient to fully replicate these differences, emphasizing the complexity of the underlying mechanisms.

## Methods

*Worm strains and Maintenance*

Worms were maintained at 20°C on Nematode Growth Medium (NGM) plates containing a lawn of OP50 *E. coli* . Strains APW320 and APW321 were created by outcrossing UR236 and UR1126 with N2 to remove *him-5* background.

&nbsp;

**Table:** 

**Strain**	**Genotype**	**Source**
N2	Wildtype	CGC
*him-5*	*him-5 ( e1490 )* V	CGC
APW320	* fsIs15[Prab-3:: fem-3 (+)::outron::mCherry+Punc-122::GFP] *	UR236 (Fagan et al., 2018)
APW321	* fsIs19[Prab-3:: tra-2 (ic)::SL2::mCherry + Pelt-2::GFP] *	UR1126 (Luo et al., 2024)

&nbsp;

&nbsp;

*Hypoxic Survival Assay*

Fifty to sixty day 1 adult worms were picked and placed on seeded NGM plates. The plates were then placed in a hypoxic chamber with oxygen levels below 40 PPM oxygen and maintained at 25°C for 18.5 hours. Following hypoxic exposure, the plates were removed from the chamber and incubated at 20°C for 24 hours to allow surviving worms to recover. After the recovery period, surviving worms were counted, with those responding to a nose touch counted as alive.

&nbsp;

*O _2_ On/Off Assay*

Fifty to seventy-five day 1 adult worms were placed on OP50 *E. coli* -seeded 60 mm plates and allowed to acclimate for 1 hour. After the acclimation period, worms were transferred to a chamber with no gas flow. Image sequences were recorded with Ximea xiQ USB 3.0 SuperSpeed camera at 1 frame per second. Microscope magnification was set to 1.5x, with the bacterial lawn as the sole focus in the field of view. Recording began with a five-minute baseline period to measure worm speed. Following the baseline recording, nitrogen gas (Airgas NF300) was introduced into the chamber at a flow rate of 0.8 L/min for seven minutes. After nitrogen exposure, the gas flow was switched to air (Airgas CGA346) at the same flow rate (0.8 L/min) for an additional five minutes. The recording was then concluded. The image sequences were analyzed using the ImageJ plugin wrmtrck_v5 to extract x and y coordinates of worms over time. These coordinates were used to calculate movement speed, which was converted to microns per second.

&nbsp;

*Statistics*

All the data were analyzed by a one-way or two-way ANOVA with post hoc multiple comparison correction as indicated in the figure legends. P values&nbsp;<&nbsp;0.05 were considered significant (GraphPad Prism 11).

&nbsp;
